# Moderate-Intensity Physical Activity, Music and Art Activities Preserved Cognitive Health in Older Adults: An Argument for Social Prescribing Solution

**DOI:** 10.3389/fnagi.2021.693791

**Published:** 2021-08-16

**Authors:** Ali Arab, Gregory J. Christie, Mehrdad Mansouri, Maryam Ahmadzadeh, Andrew Sixsmith, Martin Ester, Sylvain Moreno

**Affiliations:** ^1^School of Computing Science, Simon Fraser University, Burnaby, BC, Canada; ^2^School of Interactive Arts and Technology, Simon Fraser University, Burnaby, BC, Canada; ^3^Science and Technology for Aging Research Institute, Simon Fraser University, Burnaby, BC, Canada; ^4^Department of Gerontology, Simon Fraser University, Vancouver, BC, Canada

**Keywords:** cognitive function, dementia, Alzheimer's disease, modifiable lifestyle factors, longitudinal observational studies

## Abstract

**Introduction:** Rates of dementia are projected to increase over the coming years as global populations age. Without a treatment to slow the progression of dementia, many health policies are focusing on preventing dementia by slowing the rate of cognitive decline with age. However, it is unclear which lifestyle changes in old age meaningfully reduce the rate of cognitive decline associated with aging.

**Objectives:** Use existing, multi-year longitudinal health data to determine if engagement in a variety of different lifestyle activities can slow the rate of cognitive decline as older adults age.

**Method:** Data from the English Longitudinal Study of Aging was analyzed using a quasi-experimental, efficient matched-pair design inspired by the clinical trial methodology. Changes in short-term memory scores were assessed over a multi-year interval for groups who undertook one of 11 different lifestyle activities, compared to control groups matched across confounding socioeconomic and lifestyle factors.

**Results:** Two factors, moderate-intensity physical activity and learning activities, resulted in significant positive impact on cognitive function.

**Conclusion:** Our analysis brings cognitive benefit arguments in favor of two lifestyle activities, moderate-intensity physical activity and learning activities, while rejecting other factors advanced by the literature such as vigorous-intensity physical activity. Those findings justify and encourage the development of new lifestyle health programs by health authorities and bring forward the new health system solution, social prescribing.

## 1. Introduction

The number of older adults over the age of 65 is increasing globally, which is resulting in a range of challenges for healthcare systems and providers (Lunenfeld, 2008; Christensen et al., 2009). Rates of dementia—a disease that primarily affects older adults—are projected to increase from about 40 million people currently to 125 million by 2050. No cure exists for dementia and patients require increasing levels of labor-intense care as their symptoms progress, making dementia by far the most expensive end-of-life disease to treat (Kelley et al., 2015). Fortunately, even relatively modest delays in the onset and progression of dementia can result in significant global reductions in this disease; one estimate is that the incidence of AD would be reduced by approximately 8.6%, or 9.2 million cases if the onset of the disease was delayed by just 1 year (Brookmeyer et al., 2007).

Current pharmacological treatments are ineffective at delaying the onset of various dementias (Birks, 2006; McShane et al., 2019). Most medical recommendations, therefore, focus on preventative health measures (i.e., Social Prescribing; Fancourt et al., 2020), including physical activity, proper diet, and social and cognitively stimulating activities, to maintain cognitive health (Lang et al., 2008; De Oliveira et al., 2014; Norton et al., 2014; Deckers et al., 2015). Although these recommendations are good for maintaining overall physical health, the scientific evidence for maintaining cognitive health is unclear (Christie et al., 2017). Supporting evidence comes largely from observational studies, which often cannot determine whether engagement in a particular activity (e.g., physical exercise, music) has a causal benefit on cognitive health or only a correlational association. Data from randomized control trials have established causal effect between several activities and aging brain health such as physical activity (Cheng, 2016; Ishimaru et al., 2020), meditation and music (Alain et al., 2019). However, these studies are difficult to run and can have their methodological limitations (e.g., poor cross-sectional representation) (Harada et al., 2017).

Here, we investigated a large longitudinal, cross-sectional health dataset, the English Longitudinal Study of Aging, proposing new analytical methods that minimize the effect of confounding variables. Our analysis allowed us to determine if causal inference deems engagement in certain lifestyle activities to have benefit on cognitive health as people age. Based on previous finding (Christie et al., 2017), we hypothesized that engaging in physical activity and learning activities (e.g., art and music classes) would lead to improved cognitive health outcomes.

## 2. Method

### 2.1. Participants and Sample Data

**Figure 1:**
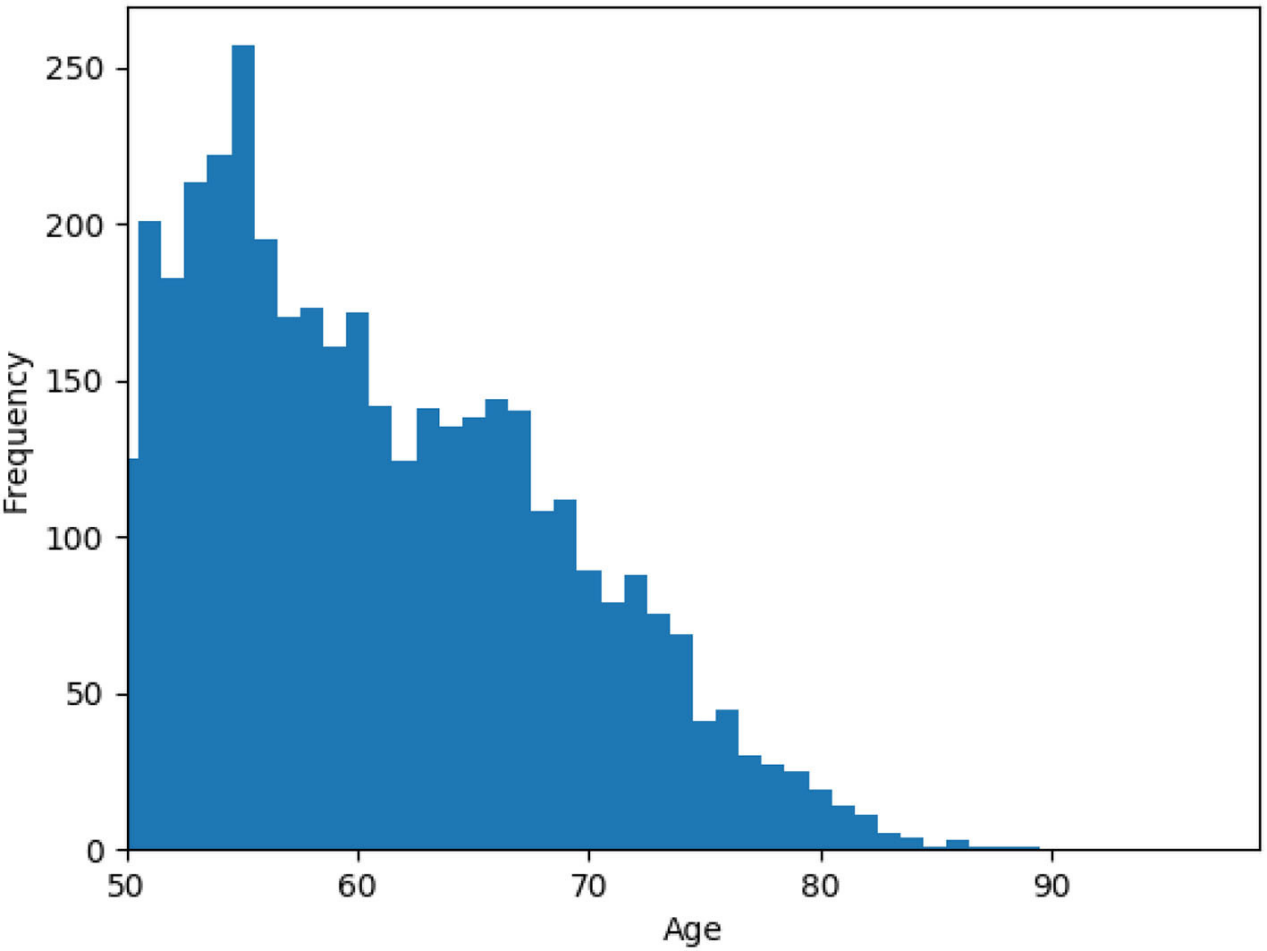
Histogram of the age variable for samples at wave 1.

We used data from the English Longitudinal Study of Ageing (ELSA), a longitudinal study of community-dwelling people aged 50 and older in England (Steptoe et al., 2013). Participants recruited using multistage-stratified probability sampling. The cohort was first assessed in 2002/2003, and was re-examined in eight examinations (waves), every 2 years. The examination dates are 2004/2005 (wave 2), 2006/2007 (wave 3), 2008/2009 (wave 4), 2010/2011 (wave 5), 2012/2013 (wave 6), 2014/2015 (wave 7), 2016/2017 (wave 8), and 2018/2019 (wave 9). For the purpose of this study, we drew 4,091 participants from waves 1–7 that did not have a missing value in their key variables. Figure 1 shows the age distribution of samples at baseline, wave 1. The statistics for other demographics, social engagement, physical activity, and cognitive function measures are reported in Table 1. Further information on the instrumentation, sampling, recruitment, and data collection procedures can be found at the ELSA's project website https://www.elsa-project.ac.uk/study-documentation.

**Table 1:** Descriptive statistics for demographics, candidate treatment variables, and the cognitive function measure used in the analysis.

	**Range**		**W1**	**W2**	**W3**	**W4**	**W5**	**W6**	**W7**
Sex	58% female								
Age	50–99	Mean	60.31						
		Std	8.35						
Socioeconomic status	1–10	Mean	6.32	6.24	6.2	6.03	5.94	6.04	5.96
		Std	2.73	2.77	2.77	2.77	2.78	2.71	2.71
Physical health	1–5	Mean	2.53	2.57	2.02	2.68	2.72	2.82	2.87
		Std	1.08	1.05	0.82	1.04	1.05	1.06	1.06
Mild physical activity	1–4	Mean	1.39	1.28	1.32	1.35	1.38	1.36	1.43
		Std	0.86	0.72	0.79	0.83	0.86	0.86	0.93
Moderate physical activity	1–4	Mean	1.62	1.59	1.6	1.68	1.76	1.8	1.88
		Std	1	0.98	1.01	1.07	1.12	1.16	1.22
Vigorous physical activity	1-4	Mean	2.93	2.95	3.01	3.03	3.1	3.13	3.21
		Std	1.26	1.25	1.25	1.24	1.21	1.21	1.18
Friendship Quality	1–4	Mean	1.87	1.88	1.83	1.86	1.88	1.87	1.8
		Std	0.78	0.76	0.75	0.76	0.76	0.75	0.73
Number of close friends	0–98	Mean	3.22	3.88	4.43	3.65	3.67	3.61	3.74
		Std	3.66	4.45	6.6	2.97	3.16	2.96	3.44
Contact frequency	1–6	Mean	2.46	2.5	2.48	2.49	2.49	2.51	3.73
		Std	1.02	1.04	1.03	1.07	1.08	1.09	0.56
Sport club membership	0–1	Mean	0.26	0.25	0.25	0.25	0.24	0.25	0.26
		Std	0.44	0.44	0.43	0.44	0.43	0.43	0.44
Church membership	0–1	Mean	0.23	0.24	0.23	0.24	0.23	0.24	0.24
		Std	0.42	0.43	0.42	0.43	0.42	0.42	0.43
Social club membership	0–1	Mean	0.2	0.2	0.19	0.2	0.2	0.19	0.19
		Std	0.4	0.4	0.39	0.4	0.4	0.39	0.39
Learning activities	0–1	Mean	0.18	0.16	0.15	0.14	0.15	0.15	0.15
		Std	0.39	0.37	0.36	0.35	0.36	0.36	0.36
Smoking	0–60	Mean	1.78	1.49	1.3	1.17	1.05	0.92	0.78
		Std	5.67	5.2	4.78	4.66	4.31	4.04	3.72
Memory index	3–24	Mean	14.55	14.88	14.93	14.76	14.62	14.59	13.95
		Std	3.17	3.21	3.34	3.38	3.54	3.65	4.04

The outcome variable to assess changes in cognitive function over time was memory index (MI), a computed variable within ELSA comprising three memory subtests on immediate, delayed, and prospective memory. MI ranges from 0 to 27, with 0 zero representing the lowest performance.

Candidate variables were selected a priori to explore causal relation with the outcome variable. Candidate variables included mild/moderate/vigorous physical activity, group membership, tobacco use, contact frequency, friendship quality, and number of close friends. Mild/moderate/vigorous physical activity variables were measured using a 4-point scale (range “more than once a week” to “hardly ever”). Examples of mild activities included laundry and home repairs; moderate intensity activity included gardening, cleaning the car, walking at moderate pace, dancing, and stretching exercises; vigorous intensity included running/jogging, swimming, cycling, aerobics/gym workout, tennis, and digging with a spade. Group membership variables asked the participants to indicate if they are a member of any sports club, social club, church, and if they participate in any learning activities. In particular, learning activities refer to participation in any education, art, or music groups, or evening classes. The values for these measures are 0 and 1 corresponding to No and Yes. Tobacco use was assessed by asking the respondent how many cigarettes they smoke per weekday. To measure contact frequency, respondents were asked how often they meet up with their friends, using a 6-point scale (range “Three or more times a week” to “Less than once a year or never”). Friendship quality captures the perceived social support by the respondents; they were asked how much they think their friends understand their feelings, using a 4-point scale (range “a lot” to “not at all”). As another measure of social support, respondents were asked how many of their friends they would say they have a close relationship with.

Confounding variables are mediator variables that affect both a given candidate variable (which is suspected to be causal) and the outcome variable. Failing to control for confounding variables may result in a misleading correlation, i.e., a possibly strong correlation that is not due to some causal relationships, damaging the validity of the analysis (Spirtes et al., 2000). The confounding variables controlled for in this study include general health and demographics. General health measures the overall health of the participants; The respondents were asked how their health is in general, using a 5-point scale (range “very good” to “very bad”). The demographics used in this study are age, gender, and socioeconomic status. Socioeconomic status is a derived variable computed based on other collected economic variables which indicate the decile of the net total wealth.

### 2.2. Data Analyses

We employed a Quasi-experimental design (QED) based on matched pair design to study the effect of candidate variables on MI. In this design, samples are paired randomly such that one sample has been exposed to a potential cause *X*_*i*_, known as the treatment, and the other sample has not been exposed to it, while both samples are constrained to be similar with respect to the other potential causes and confounders (Kuhn, 1955; Miettinen, 1968; Mansouri et al., 2018). These constraints guarantee that the effect of the other observed variables on the outcome is controlled, and the random pairing minimizes the effect of latent (unobserved) variables on the outcome.

**Figure 2:**
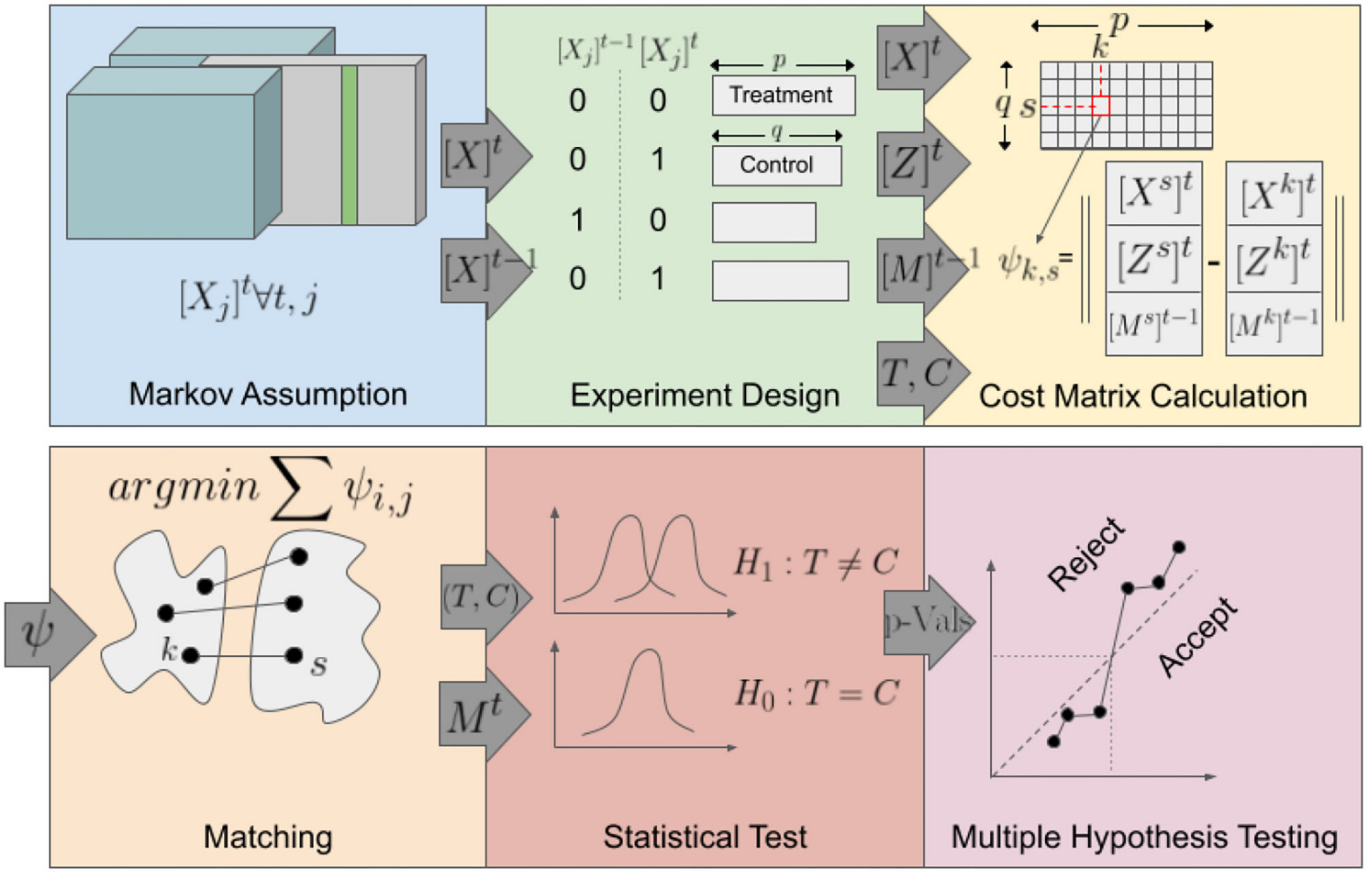
Overview of the method; schematic overview of the steps involved in the QED.

Figure 2 provides an overview of the analysis method. (i)The method iterates over all candidate variables, for each wave. (ii) Experimental Design: The treatment and control groups were defined based on the value of the candidate variable for the current and the two previous waves. (iii) Cost Matrix Calculation: A cost matrix was calculated, specifying the similarity between each pair of samples from the treatment and control groups. (iv) Matching: A set of matchings were established such that the total dissimilarity cost between matched pairs was minimized while constraining the base memory index to be equal for each pair. If for a treatment sample, two or more control samples have the same minimum distance, one was chosen randomly. (v) Sets of pairs obtained from each wave were merged, and a statistical test was performed to determine the significance of the mean difference of the outcome variable between the treatment and the control cases of the matched pairs. (vi) Finally, after computing the *p*-values for each variable and each wave, all computed *p*-values were adjusted for multiple hypothesis tests (Benjamini and Hochberg, 1995). Details of the method are provided in the following subsections.

### 2.3. Preprocessing

In order to prepare the data for the matched pairs design, a series of pre-processing operations was performed. Variables were normalized to their standard score, with the mean and standard deviation estimated across all samples and all waves. A binary version of the candidate variables *X* was generated in order to efficiently distinguish between the treatment and control cases. For continuous variables, namely the number of close friends and the number of cigarettes smoked per weekday, based on domain knowledge and variable nature, two thresholds were selected; all values above the higher threshold were converted to one and all values below the lower threshold were converted to zero. For the discrete variables, only one threshold was used for binarization.

### 2.4. Identifying Treatment and Control Groups

To capture the change in cognitive health over time, we consider the difference in MI from one wave to the next one as the outcome. Hence, the treatment group for a candidate variable in a particular wave is defined as samples where the candidate variable is zero at the initial wave and one for the two consecutive waves. Similarly, the control group consists of samples where the candidate variable is zero for both the previous wave and the current wave. The reason for defining one treatment and control group per wave is to control the impact of time-dependent latent variables such as age and social scale events.

### 2.5. Pairing

In this step, samples from treatment and control groups are paired together. To control the effect of confounding variables, the features of each pair are constrained to have a minimum distance. A hard constraint was imposed on the base memory index to make them equal for the treatment and control pairs. A sample is represented by a vector consisting of the other potential causes and the confounding variables. The distance between two samples is defined as the Euclidean distance of their corresponding vectors. In cases where multiple pairings with the same distance are possible for a sample, one is randomly selected to add the randomness component to the matched pairs design. Once the distance between all pairs is calculated, Hungarian algorithm (Kuhn, 1955) is applied to get an optimal pairing.

To define the pairing process formally, we introduce the following notation; [*Y*^*i*^]^*t*^(1 ≤ *t* ≤ *T*, 1 ≤ *i* ≤ *N*) is the target value of sample *i* in wave *t*, where *T* is the number of time points, and *N* is the number of samples; ${\left[X_{j}^{i}\right]}^{t}\left(1\leq t\leq T,1\leq i\leq N,1\leq j\leq M\right)$, is the *jth* candidate variable of sample *i* in wave *t*, where *M* is the number of candidate variables; and ${\left[Z_{j}^{i}\right]}^{t}\left(1\leq t\leq T,1\leq i\leq N,1\leq j\leq K\right)$, is the *j*^*th*^ confounding variable of sample *i* in wave *t*, where *K* is the number of confounding variables.

As the input, the Hungarian algorithm takes the cost matrix specifying the cost of each pairing. Output of the algorithm is a matching where the total cost is minimized. As usually the size of treatment and control groups are different, the number of matched pairs is the minimum size of treatment and control group. The more similar a pair, the lower the cost. The Hungarian algorithm solves the pairing of treatment and control cases as an Assignment problem:

(1) $$\begin{array}{l} u^{*}={\mathrm{argmin}}_{u}\sum_{n_{t}}\sum_{n_{c}}D\left(i,i^{\prime },t\right)u_{ii^{\prime }}\\ suchthat\;u_{ii^{\prime }}\in \left\{0,1\right\}\;\text{and }\sum_{i=1}^{n_{t}}u_{ii^{\prime }}=1\;\text{and }\sum_{i^{\prime =1}}^{n_{c}}u_{ii^{\prime }}=1 \end{array}$$

where u^*^ is the optimal assignment, *n*_*c*_ is the number of control samples and *n*_*t*_ is the number of treatment samples. In this formulation, $u_{ii^{\prime }}=1$ indicates that sample *i* of treatment set and sample *i*′ of control set are paired, and $u_{ii^{\prime }}=0$ indicates otherwise. *D*(*i, i*′, *t*) is the matching distance between treatment sample *i* and control sample *i*′ at time *t*, for variable *v* is composed of three components:

(2) $$\begin{array}{l} D\left(i,i^{\prime },t,v\right)=D_{\text{confounders}}\left(i,i^{\prime },t\right)+D_{\text{treatments}}\left(i,i^{\prime },t,v\right)\\ \;+\beta *D_{\text{target}}\left(i,i^{\prime },t\right) \end{array}$$

where β is the weight given to the target variable during the matching. The reason for allocating a component for each of the confounders, treatment, and target variable is that a high number of variables in one category does not overshadow the variables in other categories. A high weight is used for *D*_target_ to make sure that the two matched samples have similar conditions at the baseline in terms of the target variable. The subcomponents are defined as following:

(3) $$\begin{array}{l} D_{\text{target }}\left(i,i^{\prime },t\right)={\left[Y^{i}\right]}^{t-2}-{\left[Y^{i^{\prime }}\right]}^{t-2}\\ D_{\text{Confounder }}(i,i^{\prime },t)=\sum_{j=0}^{K-1}\left({\left[Z_{j}^{i}\right]}^{t}-{\left[Z_{j}^{i^{\prime }}\right]}^{t}\right)\\ D_{\text{treatment }}(i,i^{\prime },t,v)=\sum_{\begin{array}{l} j=0\\ j\neq v \end{array}}^{M-1}\left({\left[X_{j}^{i}\right]}^{t}-{\left[X_{j}^{i^{\prime }}\right]}^{t}\right) \end{array}$$

### 2.6. Statistical Test

Considering that the values for the outcome are continuous and skewed, the potential causes are binary, and the samples are paired, the Wilcoxon Signed-Rank test is used to evaluate the impact of a potential cause. The Wilcoxon Signed-Rank test for our QED evaluates the null hypothesis that there is no significant difference in the change of MI because of the value of the candidate variable. This provides us with one *p*-value for each candidate variable and each wave.

### 2.7. Adjusting the *P*-Values

Evaluating the causality of multiple candidate variables for MI produces a large number of hypotheses. This increases the chance of some of these hypotheses being incorrectly accepted by chance, which is known as the multiple comparisons problem. A standard approach to counteract the multiple comparisons problem is to adjust *p*-values and the threshold to accept them based on the false discovery rate. We use the Benjamin-Hochberg adjustment method for this purpose.

The outline of our approach is presented is Algorithm 1. The source code is made publicly available to facilitate the usage and reproducibility of the experimental results^1^.

**Algorithm 1:**
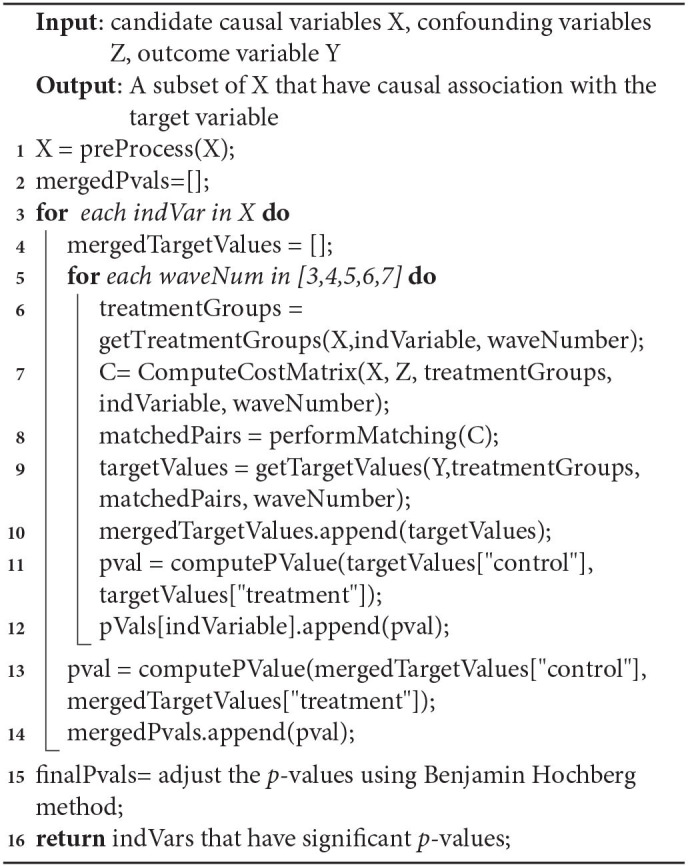
Causal Discovery Algorithm.

## 3. Results

The set of *p*-values for each combination of one variable and one wave and also for each variable in the merged population is provided in Table 2. The *p*-value for individual waves was computed based on the treatment and control group identified at each wave, whereas the last column shows the *p*-value for the merged population across all five waves. The sample sizes used to compute the *p*-values are presented below each variable.

**Table 2:** *P*-values and sample sizes associated with hypothesis testing across all waves and merged population.

**Variable**		**Wave 3**	**Wave 4**	**Wave 5**	**Wave 6**	**Wave 7**	**Merged**
Mild physical activity	*p*-value	0.4962	0.8931	0.299	0.8187	0.9885	0.57589
	*N*	42	38	68	65	71	284
Moderate physical activity	*p*-value	0.2108	0.0099	0.0004	0.1319	0.1584	0.00001
	*N*	113	117	79	171	199	679
Vigorous physical activity	*p*-value	0.7364	0.7865	0.6463	0.1102	0.8064	0.93253
	*N*	223	195	175	149	201	943
Number of close friends	*p*-value	0.4692	0.8062	0.1122	0.9159	0.6736	0.83818
	N	197	18	11	17	14	257
Friendship quality	*p*-value	0.0930	0.9169	0.6184	0.0542	0.9365	0.78565
	N	195	187	137	153	150	822
Social contact frequency	*p*-value	0.9325	0.9325	0.9325	0.9325	0.9325	0.93253
							
	N	209	233	211	221	13	887
Church membership	*p*-value	0.6618	0.7400	0.9119	0.9141	0.2600	0.77176
							
	N	59	32	38	36	50	215
Smoking	*p*-value	0.0686	0.2410	0.6534	0.2558	0.8125	0.89711
							
	N	21	20	11	9	13	74
Gym membership	*p*-value	0.7910	0.1725	0.3899	0.3402	0.2567	0.88873
							
	N	128	124	105	109	124	590
Social club membership	*p*-value	0.3264	0.7986	0.9106	0.1456	0.8096	0.84209
	N	114	87	94	103	94	492
Learning activities	*p*-value	0.1588	0.8668	0.0017	0.1918	0.0191	0.00030
	N		78	72	77	63	369

*We can see that the p-values for learning activities and moderate-intensity physical activity are statically significant, implying an association between the two variables and the cognitive function measure, MI. N, sample size*.

**Figure 3:**
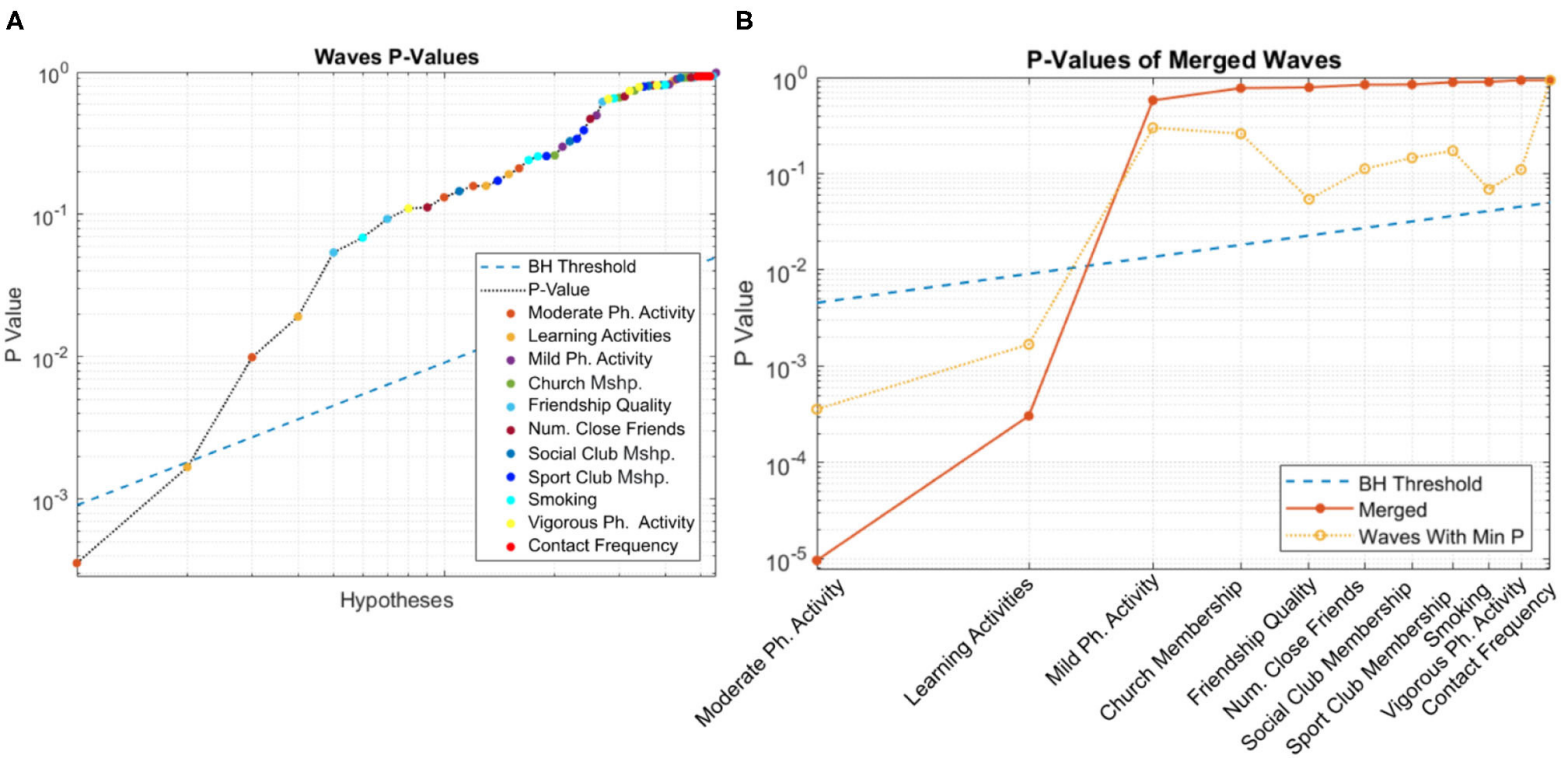
Distribution of *p*-values for **(A)** individual and **(B)** merged populations. In both cases, learning activities and moderate-intensity physical activity pass multiple hypothesis testing.

Figure 3A shows the curve corresponding to the *p*-values of candidate variables across all waves, with the blue line depicting the Benjamini-Hochberg adjusted threshold. Among the candidate variables, learning activities and moderate-intensity physical activity pass multiple hypothesis testing. The distribution of *p*-values is very healthy, with a long linear trail of evenly distributed *p*-values, as would typically be observed from random variables, and ending with few exponentially smaller *p*-values corresponding to statistically significant factors and their covariates (Oyeniran and Chen, 2016).

The corresponding curve for the merged population is shown in Figure 3B. As before, the blue line depicts the BH-adjusted 5% significance threshold. Consistent with the previous figure, both learning activities and moderate-intensity physical activity pass multiple hypothesis testing.

**Figure 4:**
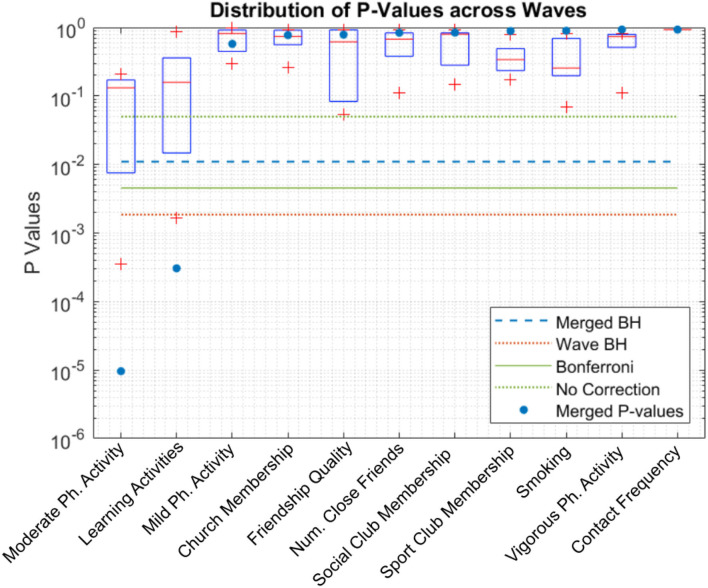
Boxplot of *p*-values of each candidate. Learning activities and moderate-intensity physical activity pass the statistical test after *p*-value adjustment even for the more conservative adjustment method, Bonferroni. “Merged BH” and “Wave BH” indicate the significance thresholds for Benjamini-Hochberg method performed on *p*-values of merged waves and individual waves, respectively.

Figure 4 shows the boxplots of *p*-values for each candidate variable. Horizontal lines depict various thresholds for rejecting the null hypothesis without correction for familywise error, Bonferroni correction (Oyeniran and Chen, 2016) and Benjamini-Hochberg correction. Learning activities and moderate-intensity physical activity remain significant even at the more conservative Bonferroni level.

**Figure 5:**
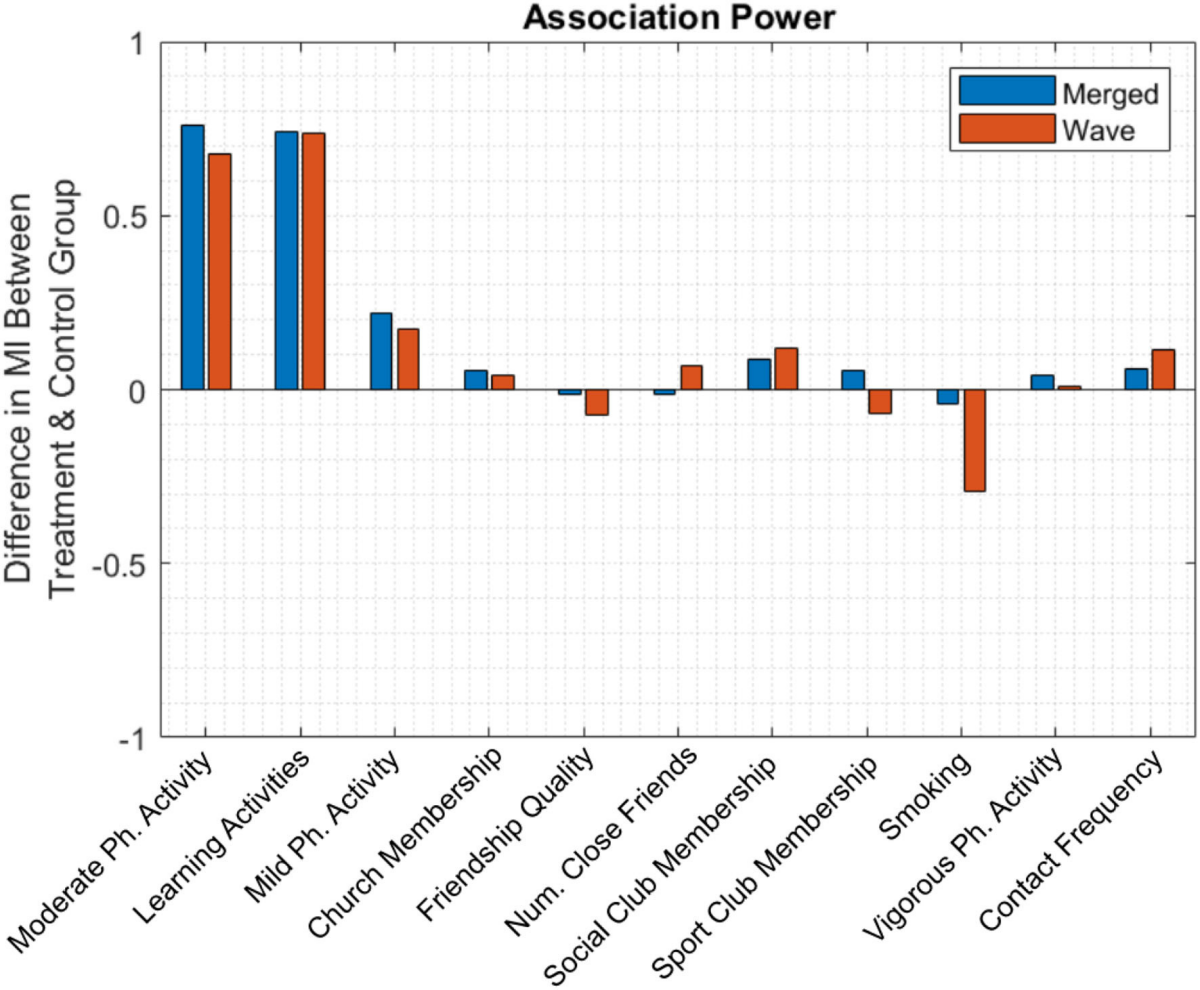
The mean difference in MI between treatment and control group. Engagement in moderate-intensity physical activity and learning activities leads to approximately 0.75 points increase in MI.

Finally, to evaluate the magnitude of the improvement expected from each treatment, we measured the mean difference in MI between treatment and control group pairs (Figure 5). Blue and red bars correspond to the merged population analysis and the per-wave analysis, respectively; in the latter case, bar height is the average of observed improvement overall five waves. For the merged population analysis, the two highest bars again correspond to the two previously identified significant candidate variables. Although significant, the benefit from each of these activities was relatively modest, leading to an improvement in MI of approximately 0.75 points on a 27-point scale.

In the per-wave analysis, smoking had a relatively large negative value, suggesting that MI decreased as the number of cigarettes smoked increased. However, due to a relative lack of participant data, this comparison lacks statistical power and was not identified as a statistically significant factor.

## 4. Discussion

For many years, researchers have sought to ascertain whether certain lifestyle changes (e.g., physical activity) in later life can preserve cognitive health during aging. Here, our findings settle part of this debate by analyzing a large, longitudinal study of aging containing detailed information on cognitive function and real-world setting engagement in various life activities. To date, many of the existing studies of lifestyle effect on cognitive health have focused on correlational changes in cognition over time (Sona et al., 2012). Inferring causation from such studies is challenging because confounding effects cannot be ruled out. Causal discovery strives to identify cause-effect relations between a set of treatment and outcome variables, where any change in a causal treatment would result in a change in the outcome (Nguyen, 2018). The gold standard method to test causality is with randomized control trials, but RCT studies are notoriously difficult to conduct in the field of longitudinal cognitive health. QED is a competent replacement for randomized controlled trials in the evaluation of causal hypotheses using observational data when the sample size is not a limiting factor. QEDs are designed to deal with the lack of random assignment in observational data by sampling from the population such that the impact of selection bias and confounding is minimized. In this study, we adopted a QED based on matched pair design to investigate the effect of undertaking and maintaining changes in various lifestyle factors and how these factors influenced long-term cognitive health.

The results of these analyses revealed only two lifestyle activities that led to improved long-term cognitive outcomes: moderate-intensity physical activity and engagement in learning activities, such as education, art, music groups, or evening classes. Specifically, older adults who engaged in moderate-intensity physical activities, such as gardening and walking, for a period of at least 4 years with a frequency of at least three times per week, gained one-third standard deviations from their baseline of memory score. Similarly, older adults who participated in learning activities for at least 4 years experienced a similar level of improvement in their cognitive health.

Broadly, these findings dovetail with past studies that sought to determine how lifestyle activities affect cognitive aging. For example, a recent meta-analysis by Woods et al. (2012) determined that cognitive stimulation improves cognition in individuals with mild cognitive impairment and moderate dementia over and above any benefit from medication. Likewise, studies of same-sex twins found that participation in leisure activities during early and middle adulthood—and participation in intellectual-cultural activities in particular—was associated with a reduced risk of Alzheimer's disease (Crowe et al., 2003).

In the present study, other lifestyle activities previously proposed as beneficial for healthy cognitive aging, such as active social participation and vigorous-intensity physical activity, did not pass hypothesis testing. We do not rule out the possibility that these and other activities may also have a positive effect on cognitive aging, which was not observed in the present study given certain limitations outlined below. Likewise, several additional issues remain to be investigated, including whether cognitive benefits are affected by the vigor or frequency of learning activities; whether exercise and learning activities are tapping into overlapping or separable neurcognitive circuitry; the added cognitive health benefit, if any, of continued engagement across longer time scales; of engagement in combinations of multiple life activities; of the duration of the cognitive protective factor following discontinuation of a given life activity; and understanding how these cognitive benefits change across older adults' lifespans.

A critical piece of converging evidence lies in determining the neuroanatomical effects of lifestyle activities and how these affect brain structure and function over time. Moderate exercise and learning activities likely both recruit wide-scale neuronal involvement for a range of activities that can include movement-related learning and delicate motor regulation. We consider it plausible that exercise and learning activities induce neuroplastic changes in the brain that counter age-related cognitive decline. Similar neuroplastic explanations have been advanced to account for cognitive improvements in other pathological populations (Särkämö et al., 2014, 2016) and healthy populations (Hötting and Röder, 2013; Moussard et al., 2016; Alain et al., 2019) and may be instantiated through a combination of increased synaptic and dendritic receptors, and changes in neuronal growth factors (Kraft, 2012; Hötting and Röder, 2013; Moreno and Bidelman, 2014); for reviews of the neurobiology of plasticity in aging see Freret et al. (2015) and Gelfo et al. (2018). Future studies will help clarify whether moderate exercise and learning activities are tapping into separable underlying mechanisms or whether these activities (and others) are accessing a unified underlying mechanism that can promote healthy cognitive aging.

As with any longitudinal study, our analysis suffers from a number of limitations. First, the data in this study was self-reported and retrospective, and could therefore be different from participants' actual behaviors. This can be exacerbated when the measurement techniques change over time, leading to a wrong inference of having found a causal relation while a developmental change actually occurring. Second, the study design included only individuals who participated in all seven waves of the ELSA (to mitigate attrition bias) and who engaged in a new lifestyle activity for at least two subsequent waves (to demonstrate a treatment effect). This may limit the extent to which the present findings apply to the broader public and may have increased the incidence of Type-II error. To increase generalizability, further replication studies with a wider variety of data sources will be required.

Nevertheless, the present results represent an important, causal discovery analysis on a large, longitudinal population. They confirm the significance of the relationship between moderate-intensity physical activities, learning activities, and long-term cognitive functioning. They also largely rule out important alternatives (i.e., spurious correlations) such as engaging in greater levels of physical activity and community classes simply because they have greater cognitive health to do so. We encourage additional research in this space and the development of community health guidelines to promote healthy cognitive aging.

## 5. Conclusion

Typical onset of dementia is at approximately age 80, and with the oldest of the baby boom generation approaching this age, there is a narrowing window with which to proactively maintain cognitive health. The results from the present study brings an answer to the baby boom generation's key question, engagement in moderate-intensity physical activity and learning activities may provide modest but significant protection against cognitive decline in aging. Therefore, we recommend the mobilization of this knowledge into community initiatives aimed at older adults and developing health policy encouraging programs such as social prescribing.

## Data Availability Statement

Publicly available datasets were analyzed in this study. This data can be found at: https://www.elsa-project.ac.uk/.

## Author Contributions

AA had full access to all of the data in the study and takes responsibility for the integrity of the data and the accuracy of the data analysis. ME and SM: study concept and design. AA, GC, and MM: drafting of the manuscript. AA and MM: statistical analysis. ME, SM, and GC: obtained funding. MA: administrative and technical support. ME and SM: study supervision. All authors acquisition, analysis, or interpretation of data, critical revision of the manuscript for important intellectual content. All authors provided input into the design, edited and revised the manuscript, and read and approved the final manuscript.

## Conflict of Interest

The authors declare that the research was conducted in the absence of any commercial or financial relationships that could be construed as a potential conflict of interest.

## Publisher's Note

All claims expressed in this article are solely those of the authors and do not necessarily represent those of their affiliated organizations, or those of the publisher, the editors and the reviewers. Any product that may be evaluated in this article, or claim that may be made by its manufacturer, is not guaranteed or endorsed by the publisher.

## Footnotes

^1^https://github.com/causal-discovery-elsa/causal_discovery_elsa.
